# Severe Scrub Typhus Presenting With Multiorgan Dysfunction and Atypical Features: Two Case Reports From the Maldives

**DOI:** 10.7759/cureus.95842

**Published:** 2025-10-31

**Authors:** Kyaw Zin Aung, Ei Ei Cho, Naw Eh Law Saw, Phyo Si Bo, Cherry Myint

**Affiliations:** 1 Internal Medicine, Kulhudhuffushi Regional Hospital, Kulhudhuffushi City, MDV; 2 Emergency Medicine, Kulhudhuffushi Regional Hospital, Kulhudhuffushi City, MDV

**Keywords:** acute kidney injury, antibiotic therapy, eschar abscess, multiorgan dysfunction syndrome, pneumonia, scrub typhus

## Abstract

Scrub typhus, caused by the obligate intracellular bacterium *Orientia tsutsugamushi*, is an emerging cause of acute febrile illness with a broad clinical spectrum ranging from mild self-limited fever to life-threatening multiorgan dysfunction syndrome (MODS). We report two unusual, clinically severe cases from the Maldives illustrating the diagnostic challenges and systemic involvement characteristic of severe scrub typhus. The first case involved a 42-year-old previously healthy male who presented with fever, progressive respiratory distress, severe hyperglycemia, metabolic acidosis, and acute kidney injury initially suggestive of a diabetic emergency. Further evaluation revealed multiorgan involvement, including pulmonary infiltrates, hepatic dysfunction, and profound renal impairment, with serological confirmation of scrub typhus. The second case described a 22-year-old male presenting with fever and a painful axillary abscess concealing a probable eschar, complicated by severe pneumonia. Both patients had pulmonary infiltrates consistent with pneumonitis and required intensive supportive care, including mechanical ventilation and renal replacement therapy. Combined antimicrobial therapy with doxycycline and azithromycin was initiated, resulting in notable clinical improvement. These cases underscore the protean clinical manifestations of scrub typhus, particularly atypical presentations and absence of classic eschar, both of which complicate early diagnosis. The significant involvement of pulmonary, renal, hepatic, and metabolic systems highlights the need for high clinical suspicion, prompt serological testing, and aggressive management in endemic regions to mitigate morbidity and mortality.

## Introduction

Scrub typhus is an acute febrile illness caused by *Orientia tsutsugamushi*, a Gram-negative obligate intracellular bacterium transmitted through the bite of larval mites (chiggers). The disease is endemic to the "tsutsugamushi triangle," which includes much of South and Southeast Asia and parts of the Western Pacific, placing over one billion people at risk and causing approximately one million cases annually [1,2]. The global burden of scrub typhus has risen in recent years, driven by climate change, ecological shifts, and improved disease recognition [1,2]. Re-emergence in previously low-incidence areas, including the Maldives, highlights its expanding epidemiological footprint [3].

Scrub typhus exhibits a broad clinical spectrum, ranging from mild febrile illness to life-threatening multiorgan dysfunction syndrome (MODS). The clinical picture may include pneumonitis, hepatitis, meningoencephalitis, myocarditis, acute respiratory distress syndrome (ARDS), and septic shock [4-7].

While eschar is classically considered a pathognomonic sign of scrub typhus, it is often absent or atypical, especially in South Asian populations. Studies suggest eschar prevalence can be as low as 7-50%, and when present, it may be missed in concealed sites or misinterpreted as abscesses or localized infections [4,8,9]. In such cases, diagnosis is often delayed or initially missed, increasing the risk of severe complications and poor outcomes [4,5].

Radiologically, pulmonary involvement in scrub typhus often manifests as bilateral alveolar opacities and interstitial infiltrates, findings that may be indistinguishable from other infectious pneumonias. These radiological features, particularly when coupled with hypoxemia, have been associated with more severe disease and serve as potential markers of poor prognosis [10]. Furthermore, when MODS ensues, mortality can exceed 24% without timely recognition and appropriate treatment [6,11]. Among these, pulmonary involvement is particularly significant, with interstitial pneumonia and bilateral alveolar infiltrates serving as predictors of severe disease and poor prognosis [9,10].

Renal involvement is a common and serious complication in severe scrub typhus. Acute kidney injury (AKI) may result from multiple overlapping mechanisms, including direct endothelial damage by the organism, systemic inflammation, hypovolemia, rhabdomyolysis, and septic shock-associated ischemic injury [6,7]. In several clinical studies, renal dysfunction has been associated with higher disease severity and increased mortality [4,9]. Electrolyte imbalances, particularly hyponatremia and hyperkalemia, are frequently observed and may necessitate renal replacement therapy in critically ill patients, as seen in one of our cases. Recognizing renal impairment early and providing timely supportive care, including dialysis when indicated, is essential for improving outcomes in such patients.

Management of scrub typhus primarily relies on antimicrobial therapy, with doxycycline as the drug of choice. However, in severe or treatment-refractory cases, combination therapy with azithromycin has shown better clinical efficacy and more rapid defervescence [11,12]. Recent evidence from the Intravenous Treatment for Scrub Typhus Trial (INTREST) supports combination intravenous doxycycline and azithromycin therapy in severe scrub typhus, demonstrating improved clinical outcomes and reduced mortality [12]. In this report, we present the following two cases of severe scrub typhus from the Maldives: one complicated by pneumonia and eschar-related abscess formation, and another mimicking diabetic ketoacidosis with MODS, including severe renal dysfunction requiring dialysis and severe pneumonia. These cases highlight the diverse and often deceptive clinical spectrum of scrub typhus, emphasizing the need for high clinical suspicion, early diagnosis, and aggressive supportive care in endemic regions.

## Case presentation

Case 1

A 42-year-old previously healthy male farmer presented to a primary health center with a one-week history of fever, productive cough, generalized myalgia, and arthralgia. Initial management included oral amoxicillin for presumptive lower respiratory tract infection. Two days later, he developed worsening shortness of breath, reduced urine output, and bilateral lower limb edema. On re-evaluation at the health center, the patient was disoriented with severe tachypnea and acidotic breathing. A random plasma glucose level of 456 mg/dL prompted a provisional diagnosis of diabetic ketoacidosis (DKA) and urgent referral to Kulhudhuffushi Regional Hospital (KRH) for further evaluation and management.

Upon arrival, the patient was obtunded, with a Glasgow Coma Scale score of 9/15, a blood pressure of 110/80 mmHg, tachypnea at 32 breaths per minute, and a heart rate of 84 beats per minute [13]. Clinical examination showed respiratory distress, bilateral coarse crepitations in the lower lung zones, elevated jugular venous pressure, bilateral lower limb edema, and deep jaundice with conjunctival injection. No eschar was identified.

**Figure 1:**
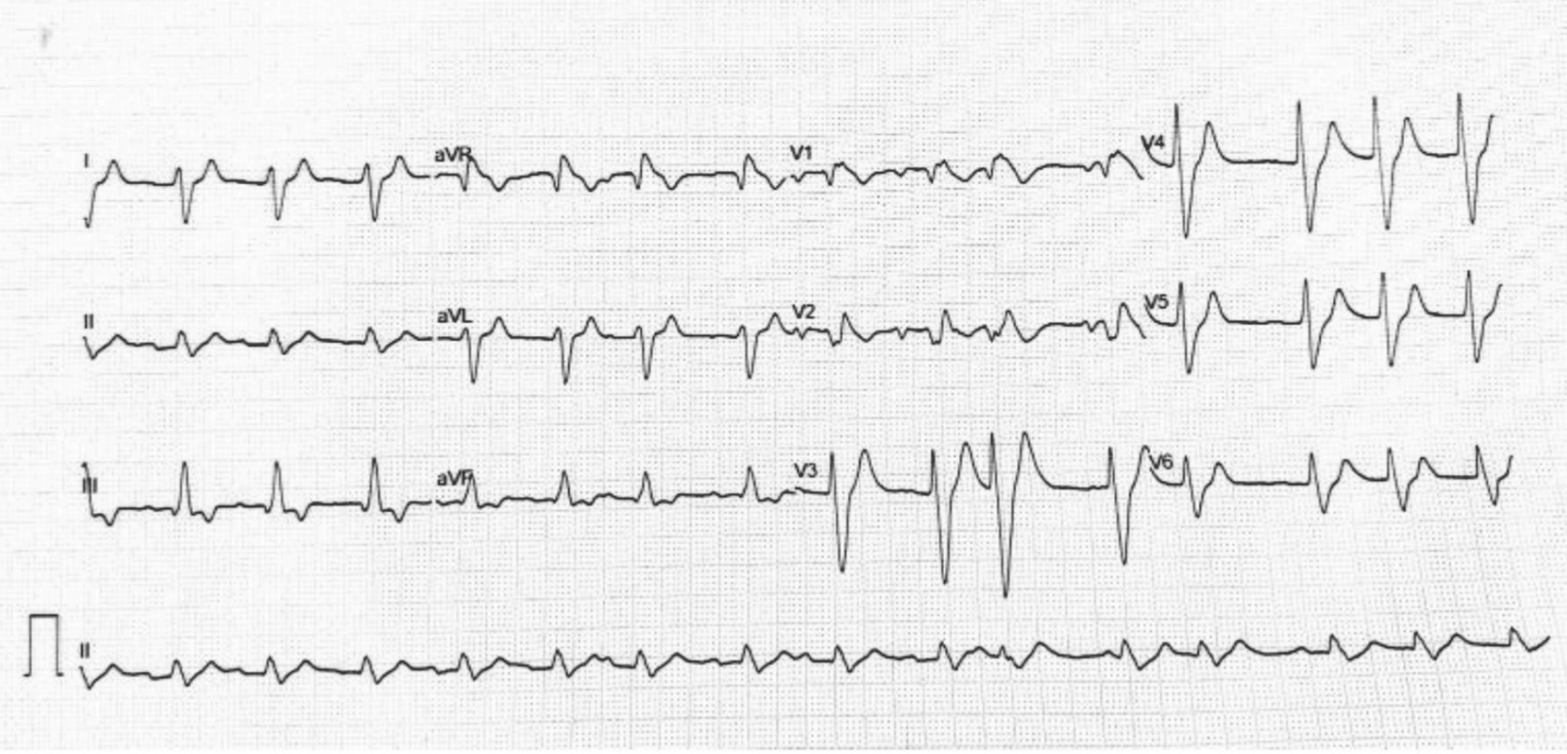
A 12-lead ECG showing classic features of severe hyperkalemia. Notable findings include tall, peaked T waves in precordial leads (V2-V5), widened QRS complexes, and absent or low-amplitude P waves.

A bedside electrocardiogram (ECG) demonstrated findings characteristic of hyperkalemia, including absence of P waves, tall, peaked T waves, and marked widening of the QRS complexes (Figure 1). Capillary blood glucose was 428 mg/dL, and glycated hemoglobin (HbA1c) was 11%, consistent with newly diagnosed, uncontrolled diabetes mellitus. Urine dipstick analysis revealed no ketones, but mild proteinuria and microscopic hematuria were present.

Arterial blood gas (ABG) revealed severe high-anion gap metabolic acidosis with marked hypoxemia, while laboratory results showed profound hyponatremia, hyperkalemia, and elevated serum creatinine, consistent with acute kidney injury. Additionally, severe hyperglycemia and elevated blood urea levels were noted, contributing to a calculated serum osmolality of approximately 292 mOsm/kg.

Urinalysis revealed a urine sodium concentration of 51 mmol/L and a urine osmolality of 272 mOsm/kg, indicating impaired renal sodium conservation and inappropriately concentrated urine in the setting of severe hyponatremia, likely due to nonosmotic antidiuretic hormone (ADH) secretion. Inflammatory markers were elevated, with neutrophilic leukocytosis and increased C-reactive protein, supporting ongoing systemic inflammation. Liver function tests were deranged, consistent with hepatic involvement, although the coagulation profile remained within normal limits. Cardiac biomarkers revealed a troponin I level of 0.0234 ng/mL, which is within the reference range (<0.03 ng/mL), effectively excluding acute myocardial injury. Serum creatine kinase (CK) was 55 U/L, at the lower limit of normal, with no evidence of significant muscle damage.

**Figure 2:**
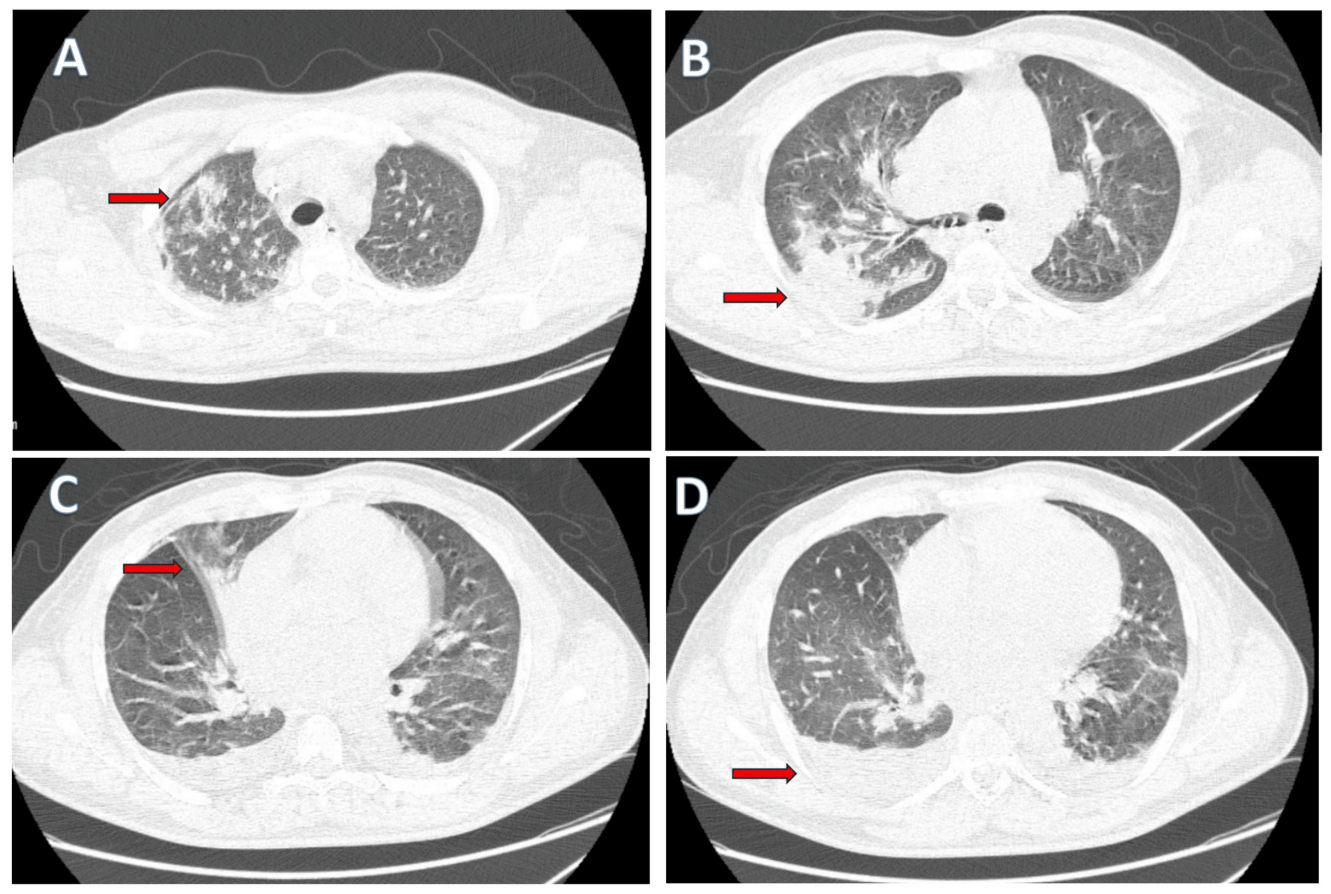
HRCT of the chest showing bilateral pulmonary involvement in a patient with severe scrub typhus pneumonia. (A) Axial HRCT at upper lung zone demonstrating bilateral patchy alveolar opacities, predominantly in the right upper lobe (red arrow). (B) Middle lung zone showing extensive bilateral ground-glass opacities and consolidation, more prominent in the right middle lobe (red arrow). (C) HRCT image at mid-lung fields reveals segmental consolidation with peribronchial thickening and interstitial infiltrates in the right lung (red arrow), indicating progression of pneumonitis. (D) Lower lung zones showing bilateral mild pleural effusion (red arrow). HRCT: high-resolution computed tomography

Serology for tropical infections revealed a positive IgM antibody for *Orientia tsutsugamushi*, confirming scrub typhus. Chest radiography (anteroposterior view) showed bilateral patchy interstitial infiltrates and areas of consolidation, primarily in the middle lung zones, more prominent on the right. High-resolution computed tomography (HRCT) of the chest demonstrated bilateral diffuse alveolar opacities, predominantly affecting the right upper and middle lobes and the left upper lobe, with air bronchograms and bilateral pleural effusions, indicating extensive pulmonary involvement (Figures 2A-2D). Abdominal ultrasound demonstrated mild ascites and bilateral small pleural effusions.

These findings, in the context of confirmed scrub typhus infection, were consistent with multiorgan dysfunction syndrome (MODS) involving renal, hepatic, pulmonary, and metabolic systems. Emergency management was initiated in accordance with established protocols for life-threatening electrolyte and acid-base disturbances. This included intravenous calcium gluconate to stabilize cardiac membranes, insulin infusion, and nebulized salbutamol to facilitate intracellular potassium shift in the treatment of hyperkalemia, intravenous sodium bicarbonate for severe metabolic (lactic) acidosis, and 3% hypertonic saline for symptomatic hyponatremia. Due to worsening respiratory failure and decreased consciousness, endotracheal intubation was performed. One session of urgent hemodialysis was initiated for life-threatening renal failure and electrolyte disturbances.

The patient was admitted to the intensive care unit (ICU) for close monitoring and advanced supportive care. Intravenous azithromycin was started in combination with oral doxycycline (administered via nasogastric tube) for scrub typhus, alongside variable-rate insulin infusion for glycemic control. Intravenous hydrocortisone was administered as adjunctive therapy due to a slow clinical response and persistent renal dysfunction. Intermittent hemodialysis was continued during the ICU stay.

**Figure 3:**
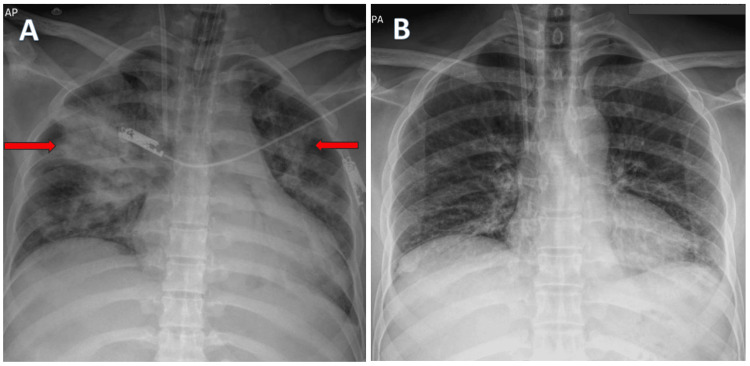
Serial chest radiographs showing pulmonary involvement and gradual radiological improvement following antibiotic therapy in severe scrub typhus. (A) Chest radiograph on admission showing bilateral patchy alveolar infiltrates (red arrows), consistent with scrub typhus pneumonitis. (B) Serial chest X-ray on day seven of hospitalization showing marked resolution of infiltrates, consistent with radiological improvement following antimicrobial therapy and supportive care.

The patient demonstrated gradual clinical improvement by day seven of hospitalization with aggressive organ support and targeted antimicrobial therapy. By day 14, following completion of a full 14-day course of combined antibiotic therapy, clinical condition, laboratory parameters, and radiologic findings showed significant improvement. Key laboratory trends observed during hospitalization are summarized in Table 1, and serial chest radiographs demonstrating progressive resolution of pulmonary infiltrates are shown in Figures 3A, 3B. The patient was subsequently discharged in stable condition.

**Table 1:** Summary of laboratory investigations at admission and on subsequent follow-up days. Day 1 refers to the day of admission; subsequent days follow accordingly. Hb: hemoglobin; Na^+^: sodium; K^+^: potassium; ALT: alanine aminotransferase; AST: aspartate aminotransferase; ALP: alkaline phosphatase; Cr: creatinine; BUN: blood urea nitrogen; PaO_2_: partial pressure of oxygen; PaCO_2_: partial pressure of carbon dioxide

Parameters	Day 1	Day 2	Day 3	Day 4	Day 5	Day 6	Day 7	Day 10	Day 14	Reference range
Hb (g/dL)	11.2	10.6	10.8	9.8	10.1	9.7	9.5	9.8	9.9	10.6-13.5
WBC (×10⁹/L)	28.19	13.1	19.58	18.59	21.86	14.67	11.28	7	8.5	4-11.5
Platelet (×10⁹/L)	380	320	291	380	420	266	341	197	203	150-450
CRP (mg/L)	78	71.1	74.8	68.4	79	42	27.7	12.4	6	<10
Serum Na^+^ (mmol/L)	101	120	122	130	131	135	132	137	139	135-145
Serum K^+^ (mmol/L)	7	5.2	4.8	4.6	4.9	4.8	4.4	3.9	3.9	3.5-5.5
Total bilirubin (mg/dL)	14.7	11.6	10.9	7	6.2	4.3	2.3	1.7	1.4	0.2-1.3
ALT (U/L)	154	116	106	89.9	77	60	53	42	39	<35
AST (U/L)	74	90	73	52	49	37	42	40	40	14-36
ALP (U/L)	460	425	437	376	496	435	368	368	258	38-126
Serum creatinine (mg/dL)	10.8	8.1	9	7.9	9.4	7.5	5.4	3	1.5	0.52-1.04
BUN (mg/dL)	85	59.8	77.1	71	91.6	73.8	78.5	70	48	9-20
pH	6.9	7.46	7.49	7.44	7.43	7.49	7.5	7.42	7.42	7.35-7.45
PaO_2_ (mmHg)	45	73.1	80.8	72	108	109	110	106	109	83-108
PaCO_2_ (mmHg)	27.6	25.7	29.6	29.8	35.5	33.7	32.2	34.3	38.8	32-48

**Figure 4:**
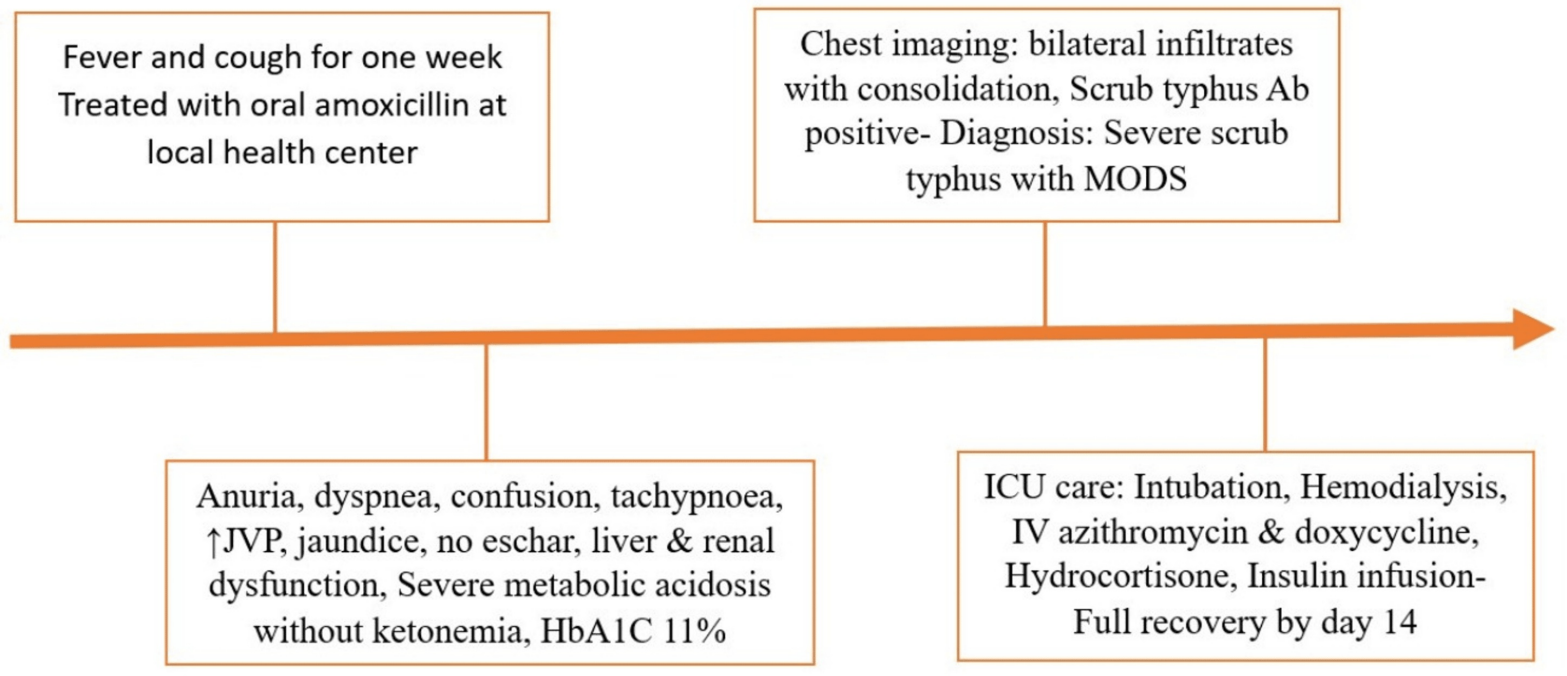
Clinical course of severe scrub typhus with MODS. JVP: jugular venous pressure; MODS: multiorgan dysfunction syndrome

At outpatient follow-up, the patient remained clinically stable, with normalization of renal function, liver enzymes, and glycemic parameters. Pulmonary findings had resolved on repeat imaging. The patient reported residual fatigue but no recurrence of symptoms. This case highlights the rare but life-threatening presentation of scrub typhus with multiorgan dysfunction, emphasizing the importance of early recognition and comprehensive supportive management, especially in resource-limited settings. The overall clinical course, including key interventions and recovery milestones, is illustrated in Figure 4.

Case 2

**Figure 5:**
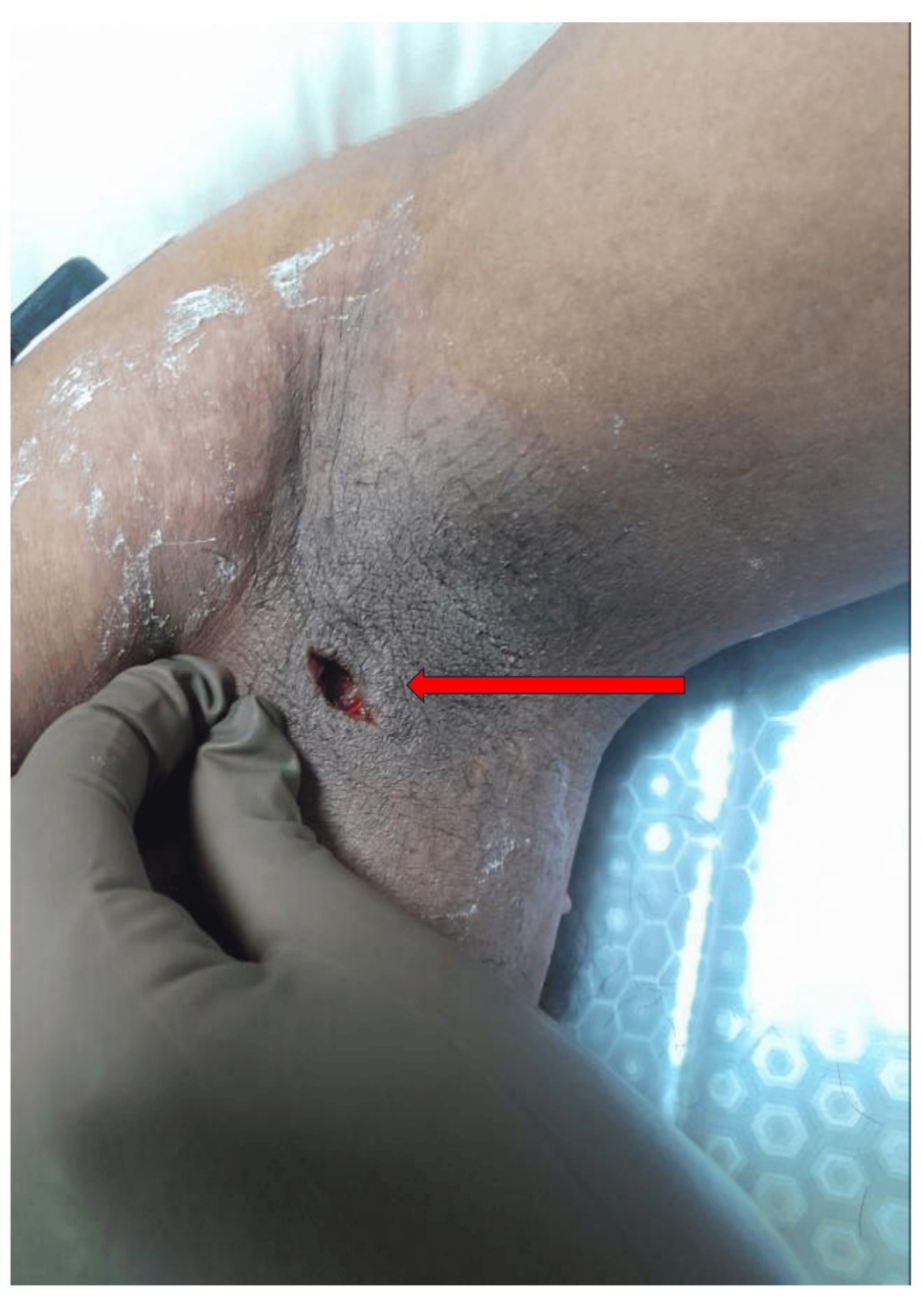
Post incision and drainage site of a left axillary abscess at the location of a scrub typhus eschar. The red arrow indicates the healing wound cavity.

A 22-year-old previously healthy male presented to a primary health center with a three-day history of fever, productive cough, and a painful swelling in the left axilla. Clinical examination revealed a left axillary abscess, which was incised and drained under aseptic conditions and local analgesia (Figure 5). Subsequently, the patient developed acute shortness of breath and oxygen desaturation, prompting urgent referral to Kulhudhuffushi Regional Hospital (KRH).

On arrival at KRH, the patient was toxic-appearing and exhibited dyspnea. Vital signs included blood pressure of 110/80 mmHg, heart rate of 110 beats per minute, respiratory rate of 28 breaths per minute, and oxygen saturation of 88% on room air. Physical examination showed tachypnea, tachycardia, use of accessory respiratory muscles, and bilateral coarse crepitations over the lower lung fields.

**Figure 6:**
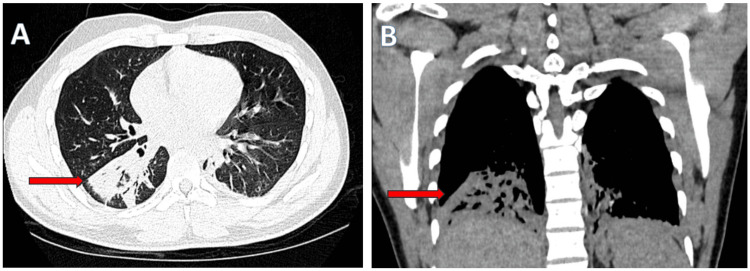
HRCT chest showing consolidation in the right lower lobe. (A) Axial and (B) coronal HRCT images of the chest showing a consolidation with air bronchograms in the posterior basal segment of the right lower lobe (red arrows). These findings are consistent with lobar pneumonia. HRCT: high-resolution computed tomography

Arterial blood gas analysis revealed moderate hypoxemia. Laboratory investigations demonstrated elevated C-reactive protein (CRP) levels and confirmed the diagnosis of scrub typhus through a positive IgM antibody by enzyme-linked immunosorbent assay (ELISA). A summary of key laboratory findings is presented in Table 2. CT chest demonstrated focal consolidation with air bronchograms in the posterior basal segment of the right lower lobe, consistent with lobar pneumonia secondary to scrub typhus (Figures 6A, 6B).

**Table 2:** Summary of laboratory investigations at admission and on subsequent follow-up days. Day 1 refers to the day of admission; subsequent days follow accordingly. Hb: hemoglobin; ALT: alanine aminotransferase; AST: aspartate aminotransferase; ALP: alkaline phosphatase; PaO_2_: partial pressure of oxygen; PaCO_2_: partial pressure of carbon dioxide

Parameters	Day 1	Day 3	Day 5	Day 7	Reference range
Hb (g/dL)	11.5	11.4	11.5	12	10.6-13.5
WBC (×10⁹/L)	13.88	8.26	8.2	9.64	4-11.5
Platelet (×10⁹/L)	428	354	327	390	150-450
CRP (mg/L)	59.14	29.8	19	8.4	<10
Total bilirubin (mg/dL)	0.6	0.6	0.6	0.8	0.2-1.3
ALT (U/L)	22	20	22	20	<35
AST (U/L)	24	18	22	22	14-36
ALP (U/L)	85	83	90	84	38-126
Serum creatinine (mg/dL)	0.8	0.9	1	0.8	0.52-1.04
pH	7.44	7.42	7.38	7.42	7.35-7.45
PaO_2_ (mmHg)	72	88	89	100	83-108
PaCO_2_ (mmHg)	31	34	35	34	32-48

Based on clinical presentation, radiologic findings, and serologic confirmation, a diagnosis of scrub typhus pneumonia with an eschar-associated axillary abscess was established. Although abscess formation is atypical in scrub typhus, this finding may reflect either a secondary bacterial superinfection at the eschar site or a rare manifestation of local tissue inflammation associated with the primary infection.

The patient was admitted to the ICU for close monitoring and treatment. Combination antimicrobial therapy with intravenous azithromycin and oral doxycycline was initiated per severe scrub typhus management guidelines, and the axillary wound was managed with alternate-day sterile dressings. Over 72 hours, the patient showed marked clinical improvement, with resolution of dyspnea, normalization of oxygen saturation, and decreased inflammatory markers. Follow-up imaging demonstrated partial resolution of pulmonary infiltrates.

He was transferred to the general medical ward and completed a seven-day course of antibiotics. The patient was discharged on day seven in stable condition, showing significant clinical, radiologic, and laboratory improvement. At follow-up, the patient showed satisfactory wound healing and a mild residual cough.

## Discussion

Scrub typhus continues to pose significant diagnostic and therapeutic challenges in endemic areas due to its protean manifestations and overlap with other tropical febrile illnesses. Both patients in this report presented with atypical or severe complications, one with multiorgan dysfunction mimicking diabetic ketoacidosis, and the other with pneumonia following an eschar-associated abscess, underscoring the complexity of clinical recognition, particularly in resource-limited settings.

The absence or atypical presentation of eschar, as seen in both cases, remains a major obstacle to early diagnosis. Although eschar is considered a classic finding, its prevalence varies geographically, with rates ranging from 7% to 50% in South and Southeast Asia [4,8,9]. In the second patient, a left axillary abscess obscured a probable eschar site, whereas in the first patient, no eschar was identified, contributing to initial diagnostic uncertainty. Eschar may be missed in darker-skinned patients or hidden in inconspicuous areas, such as the groin, axilla, or inframammary folds [8].

Pulmonary involvement in scrub typhus ranges from mild bronchitis to severe interstitial pneumonia and acute respiratory distress syndrome (ARDS), the latter being associated with poor prognosis [9,10]. Both patients demonstrated significant hypoxia and radiologic findings of bilateral alveolar infiltrates. In particular, high-resolution computed tomography (HRCT) in the first case revealed diffuse alveolar opacities with pleural effusions - hallmarks of severe scrub typhus pneumonitis. These findings align with previous literature suggesting that interstitial pneumonia is common and may serve as a marker of disease severity [9,10].

Renal involvement is another key complication of severe scrub typhus. Our first patient developed profound acute kidney injury (creatinine 10.8 mg/dL), electrolyte imbalances (hyperkalemia, hyponatremia), and metabolic acidosis requiring urgent dialysis. This presentation reflects documented renal complications arising from direct endothelial injury, systemic inflammatory response, and ischemic tubular necrosis in scrub typhus [6,7]. Acute kidney injury (AKI) has been recognized as an independent predictor of mortality and often necessitates prompt initiation of renal replacement therapy [4,9].

Both cases illustrate how the absence of a typical eschar and nonspecific clinical features can delay diagnosis, particularly in rural or resource-limited settings where rapid testing is unavailable. Such delays often contribute to disease progression and increased severity [5].

Treatment of scrub typhus remains centered on timely antibiotic therapy. While doxycycline remains the first-line treatment, emerging evidence supports the use of combination therapy with azithromycin in severe cases. The Intravenous Treatment for Scrub Typhus Trial (INTREST) demonstrated that patients with severe scrub typhus receiving intravenous doxycycline plus azithromycin had more rapid clinical recovery and fewer complications compared to those receiving monotherapy [12]. Both of our patients were treated with intravenous azithromycin and oral doxycycline, with notable clinical improvement by day seven, highlighting the potential benefit of combination therapy in critically ill patients.

Finally, aggressive supportive management, including respiratory support, hemodialysis, glycemic control, and corticosteroids, was critical in the first case. The adjunctive use of intravenous hydrocortisone was considered in the context of suspected septic shock and slow clinical response to antibiotics, consistent with some clinical protocols for managing scrub typhus-induced organ dysfunction [7].

These cases underscore the importance of maintaining a high index of clinical suspicion for scrub typhus in febrile patients presenting with multiorgan dysfunction, even in the absence of an identifiable eschar, particularly in endemic regions. Early recognition, prompt initiation of combination antimicrobial therapy, and intensive supportive care are crucial for reducing morbidity and mortality, especially in endemic areas like the Maldives.

## Conclusions

Scrub typhus continues to pose substantial diagnostic and therapeutic challenges in endemic regions, owing to its nonspecific clinical presentation, inconsistent presence of eschar, and propensity for severe complications, such as interstitial pneumonia, acute kidney injury, and multiorgan dysfunction syndrome (MODS). The cases presented highlight the diverse and severe clinical spectrum of the disease, including rare manifestations such as phenotypic mimicry of diabetic ketoacidosis with concurrent renal and respiratory failure, and eschar-associated abscess formation with severe pneumonia.

In endemic settings, a high index of clinical suspicion is essential, particularly in patients with undifferentiated febrile illness and multiorgan involvement. Timely recognition and early initiation of appropriate antimicrobial therapy, especially combination regimens, such as doxycycline and azithromycin in severe cases, are critical to reducing morbidity and preventing progression to life-threatening complications. These cases underscore the need to enhance clinician awareness, include scrub typhus routinely in the differential diagnosis of febrile illnesses with systemic involvement, and strengthen diagnostic capacity at primary and peripheral healthcare levels to enable earlier intervention and improved outcomes.
